# Patient and hospital determinants of primary percutaneous coronary intervention in England, 2003–2013

**DOI:** 10.1136/heartjnl-2015-308616

**Published:** 2016-01-06

**Authors:** M Hall, K Laut, T B Dondo, O A Alabas, R A Brogan, N Gutacker, R Cookson, P Norman, A Timmis, M de Belder, P F Ludman, C P Gale

**Affiliations:** 1 Leeds Institute of Cardiovascular and Metabolic Medicine, University of Leeds, Leeds, UK; 2 York Teaching Hospital NHS Foundation Trust, York, UK; 3 Centre for Health Economics, University of York, York, UK; 4 School of Geography, University of Leeds, Leeds, UK; 5 NIHR Biomedical Research Unit at Barts Health, Queen Mary University, London, UK; 6 The James Cook University Hospital, South Tees Hospitals NHS Foundation Trust, Middlesbrough, UK; 7 Queen Elizabeth Hospital, Birmingham, UK; 8 National Institute for cardiovascular Outcomes Research (NICOR), University College, Institute of Cardiovascular Science, London, UK

## Abstract

**Objective:**

Primary percutaneous coronary intervention (PPCI) for ST-elevation myocardial infarction (STEMI) is insufficiently implemented in many countries. We investigated patient and hospital characteristics associated with PPCI utilisation.

**Methods:**

Whole country registry data (MINAP, Myocardial Ischaemia National Audit Project) comprising PPCI-capable National Health Service trusts in England (84 hospital trusts; 92 350 hospitalisations; 90 489 patients), 2003–2013. Multilevel Poisson regression modelled the relationship between incidence rate ratios (IRR) of PPCI and patient and trust-level factors.

**Results:**

Overall, standardised rates of PPCI increased from 0.01% to 86.3% (2003–2013). While, on average, there was a yearly increase in PPCI utilisation of 30% (adjusted IRR 1.30, 95% CI 1.23 to 1.36), it varied substantially between trusts. PPCI rates were lower for patients with previous myocardial infarction (0.95, 0.93 to 0.98), heart failure (0.86, 0.81 to 0.92), angina (0.96, 0.94 to 0.98), diabetes (0.97, 0.95 to 0.99), chronic renal failure (0.89, 0.85 to 0.90), cerebrovascular disease (0.96, 0.93 to 0.99), age >80 years (0.87, 0.85 to 0.90), and travel distances >30 km (0.95, 0.93 to 0.98). PPCI rates were higher for patients with previous percutaneous coronary intervention (1.09, 1.05 to 1.12) and among trusts with >5 interventional cardiologists (1.30, 1.25 to 1.34), more visiting interventional cardiologists (1–5: 1.31, 1.26 to 1.36; ≥6: 1.42, 1.35 to 1.49), and a 24 h, 7-days-a-week PPCI service (2.69, 2.58 to 2.81). Half of the unexplained variation in PPCI rates was due to between-trust differences.

**Conclusions:**

Following an 8 year implementation phase, PPCI utilisation rates stabilised at 85%. However, older and sicker patients were less likely to receive PPCI and there remained between-trust variation in PPCI rates not attributable to differences in staffing levels. Compliance with clinical pathways for STEMI is needed to ensure more equitable quality of care.

## Introduction

Variation between and within countries in the adoption of evidence based care is a major global healthcare problem.^1^ ST-elevation myocardial infarction (STEMI) comprises 25–40% of all acute myocardial infarction cases in Europe and the USA, and represents one of the most common reasons for hospitalisation worldwide.^2–4^ For its acute management, there is a Class 1 Level A treatment recommendation for primary percutaneous coronary intervention (PPCI) because it is associated with a relative risk reduction of death of approximately 37% over fibrinolytic therapy.^5^ However, studies report substantial inter-regional differences in its utilisation.^6–11^ It is not surprising that strategies to ensure its wider and faster diffusion have been at the forefront of international efforts to improve the quality and outcomes of cardiovascular care.

Thus far none of the studies investigating the diffusion of PPCI have incorporated whole healthcare systems or assessed patient-level and hospital-level data simultaneously.^8^ ^9^ ^12^ Rather, they have been based on small samples, select cohorts, span only part of the implementation phase or have been limited through their use of non-contemporaneous, administrative or insurance-based databases.^7^ ^13–15^ This is particularly important because convenience sampling of only cases and centres with up-to-standard data may bias estimates or create uncertainty around the impact of clinical variables. In addition, earlier studies typically have been ecological in design—an approach which limits the interpretation of inferences about outcomes relating to individuals.^8^ ^9^ As a result, the literature provides an uncertain picture of the relationship between patient-level and hospital-level characteristics and the diffusion of PPCI. Many countries do not yet provide PPCI as the treatment of choice for the emergency reperfusion of STEMI.^16–20^ Contemporary knowledge about the barriers and enablers of PPCI implementation will be essential to the future effective provision of nationwide services.

In the UK, continuous whole country data for STEMI and associated PPCI are collected through the Myocardial Ischaemia National Audit Project (MINAP) registry. This provides a unique opportunity to undertake large-scale phenotype- and intervention-specific research. To address the limitations of previous studies, for a clinically important cohort, we used MINAP data to investigate the diffusion of PPCI for STEMI across the National Health Service (NHS) in England. Specifically, the aims of this study were (1) to describe between-hospital and temporal variation in standardised rates of utilisation of PPCI, and (2) to quantify patient-level and trust-level factors associated with its implementation.

## Methods

### Setting and design

Anonymised patient level data were obtained from the national heart attack register, MINAP. MINAP is a comprehensive clinical database of patients hospitalised with acute myocardial infarction, mandated by the Department of Health for all hospitals in England and Wales.^21^ Data are collected prospectively at each hospital, electronically encrypted and transferred online to a central database. Data entry is subject to routine error checking and a mandatory annual validation exercise. MINAP is overseen by a multi-professional steering group and the National Institute for Cardiovascular Outcomes Research (NICOR) executive.^22^ Each MINAP entry provides patient demographic data and clinical details of the patient journey across 122 data items. NICOR, which includes MINAP, has support under section 251 of the NHS Act 2006 to use patient information for medical research without consent (Ref: NIGB: ECC 1-06 (d)/2011). Ethical approval was not required under NHS research governance arrangements.

Individuals were eligible for inclusion in our study if they were admitted directly to a PPCI-capable hospital (a hospital with the infrastructure and skills to offer PPCI) in England with STEMI between 1 January 2003 and 30 June 2013 and were aged ≥18 years. Inter-hospital transfers were not included in the analysis, nor hospitals performing only sporadic PPCI procedures. STEMI diagnoses were identified via the discharge diagnosis as recorded in MINAP. Patient baseline characteristics were age, gender, ethnicity, comorbidities, previous medical history, and socioeconomic status (based on the 2010 English Indices of Multiple Deprivation, and categorised from most deprived (5) to least deprived (1)). Patients were selected as having received PPCI according to their initial reperfusion strategy. To be eligible for PPCI, patients met the following criteria: clinical presentation suggestive of myocardial infarction with symptom duration of ≤12 h and ST-segment elevation of ≥0.1 mV in at least two contiguous leads, or ≥0.2 mV in V1–V3, or presumed new-onset left bundle branch block on electrocardiography.

The characteristics of hospitals for each year of study, including number of interventional cardiologists, number of visiting interventional cardiologists, type of PPCI service offered (‘24/7’ or ‘9 to 5’ service), teaching status, and hospital size (measured as total number of hospital beds) were obtained from the British Cardiovascular Intervention Society (BCIS) survey (personal communication with PFL). In addition, publicly available hospital trust data concerning the total available bed days and number of occupied bed days by consultant main specialty were obtained from NHS England (http://www.england.nhs.uk/statistics/statistical-work-areas/bed-availability-and-occupancy/bed-data-overnight/) from 2003 to 2013. Although these latter data were not available for individual hospitals within a trust, among our cohort of hospitals there were only three trusts which contained more than one hospital providing PPCI services (in these cases the trust characteristics were applied to both hospitals). Euclidean (straight line) distances from each patient's residence to the nearest trust with onsite PPCI facilities were derived from patients’ and trusts’ postcodes, translated into Cartesian coordinates (see online supplementary appendix section 1). All trust data were linked to MINAP data through the NICOR analytical team before releasing pseudonymised patient and hospital identification data for the analyses.

### Outcome

The primary endpoint was PPCI utilisation, calculated as the rate of PPCI per hospital out of the total number of STEMI hospitalisations per hospital. For patients with multiple PPCIs across multiple hospitalisations, all were included in the calculation.

### Statistical analysis

Baseline characteristics were summarised as numbers and percentages, means and SDs, and medians and IQRs for categorical, normally distributed and non-normally distributed continuous data, respectively. To analyse temporal trends of PPCI utilisation, age-and sex-standardised rates of PPCI per 100 000 STEMI hospitalisations and 95% CIs were summarised by year of hospitalisation for PPCI.

We built a series of multilevel Poisson regression models (which accounted for the clustering of patients within trusts) to quantify the degree to which patient characteristics and trust-level factors were associated with annual hospital PPCI utilisation, with the number of STEMI hospitalisations as the offset term. Initially, univariable models were fitted and subsequently multivariable models built by incrementally adding variables which had significant associations with PPCI utilisation to a base model that comprised age, sex and year as well as a random intercept for each trust. The Index of Multiple Deprivation quintile did not contribute significantly to the base model and was, therefore, omitted. Initially models focused on patient characteristics and then on trust-level characteristics to assess their relative impact upon PPCI utilisation. Model estimates were presented as incidence rate ratios (IRRs) which are interpreted as the linear increase or decrease in trust PPCI utilisation over the study period after adjusting for confounding factors. All explanatory variables were introduced as fixed effects, except year, which was included as a random effect to allow for variation in the rate of change of PPCI utilisation over time between trusts. To assess the degree of variation in PPCI utilisation rates attributed to the trust level (and not explained by the model), we obtained an approximation to the intraclass correlation coefficient (ICC) from a multilevel linear regression model which used PPCI rates as the outcome (see online supplementary appendix section 1).

The extent of patients with missing data for case mix variables and confounders was evaluated and managed by multiple imputation. To undertake this, we imputed 10 datasets using chained equations before analysis and pooled estimates using Rubin's rules across the imputed datasets. Imputation models were congruent to analysis models and included the outcome variable following methods used previously for MINAP data (see online supplementary appendix table S1). To check the accuracy and consistency of the imputation process, imputed data were compared to complete case analysis (see online supplementary appendix table S2). The Poisson model was checked for overdispersion by comparison with a negative binomial model (see online supplementary appendix table S3).

Multiple imputation was performed in R V.3.1.2 (The R Project for Statistical Computing, cran.r-project.org) and all further statistical analyses were performed in Stata MP V.13 (Stata Corp, Texas, USA). Values of p<0.05 were considered to be statistically significant and all tests were two sided.

## Results

**Figure 1:**
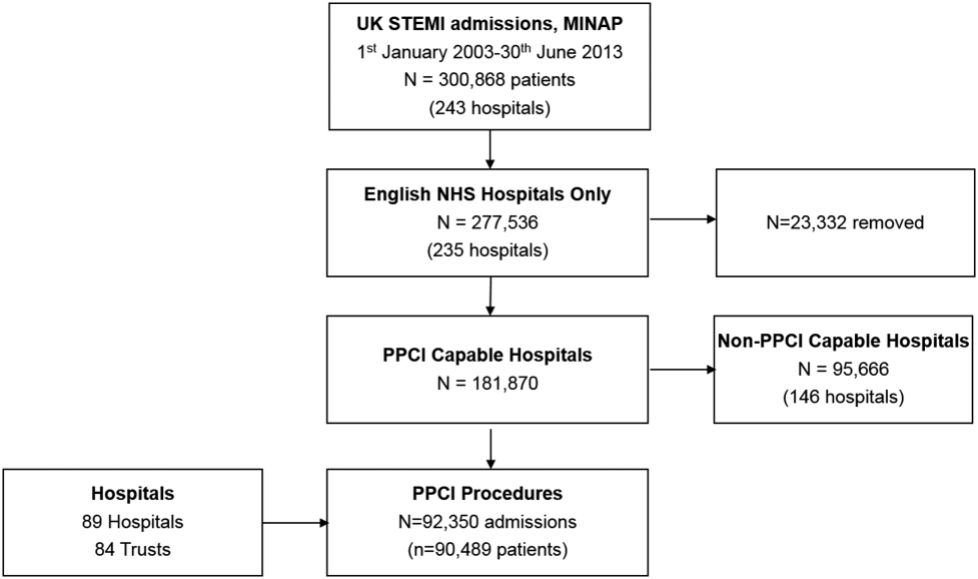
Consort diagram of exclusions of the Myocardial Ischaemia National Audit Project (MINAP) dataset. PPCI, primary percutaneous coronary angiography; ST, ST-elevation myocardial infarction.

We identified 300 868 individuals with STEMI admitted to hospitals in the UK. We excluded 23 332 (7.8%) records from hospitals outside England, and 95 666 (34.5%) records for individuals in non-PPCI capable hospitals. Of the remaining 181 870 patients with a discharge diagnosis of STEMI, across 84 trusts, there were a total of 92 350 (50.8%) hospitalisations for 90 489 patients for which PPCI was performed. There were 1859 (1%) patients who had multiple hospitalisations (figure 1).

The mean±SD age was 63.7±13.2 years; 26.4% of patients were female and 14% had diabetes. Across the study years, the mean age increased from 60.1 to 63.8 years (table 1). There was a decrease in the proportion of patients belonging to the most deprived group (56.7–22.4%), and a corresponding increase in patients belonging to the least deprived group (3.3–18.4%). Moreover, we found that the proportion of patients who received PPCI and lived further away from the PPCI centre increased from no cases living >30 km away in 2003/2004 to 18.3% of cases living >30 km away in 2011/2013. The median number of trust beds remained stable over the study period (843.5 in 2005/2007 to 837 in 2011/2013), while the mean number of cardiologists increased (1.2 in 2005/2007 to 4.9 in 2011/2013). The number of trusts performing PPCI ‘24/7’ (24 h service 7 days a week) increased from 7.2% in 2005/2007 to 57% in 2011/2013.

**Table 1:** Patient characteristics by period of admissions to hospital in England, 2003–2013

Patient demographics, history and cardiac status	Period of admission to hospital				
	Total (n=90 498)	2003–2004 (n=30)	2005–2007 (n=4299)	2008–2010 (n=35 000)	2011–2013 (n=51 169)
Age, years	63.7 (13.2)	60.1 (13.2)	63.0 (13.0)	63.6 (13.2)	63.8 (13.2)
Female	23 776 (26.4)	7 (23.3)	1119 (26.1)	9286 (26.6)	13 364 (26.3)
Ethnicity					
Caucasian	71 631 (90.0)	17 (77.3)	2999 (83.2)	27 241 (90.6)	41 374 (90.2)
Other	7941 (10.0)	5 (22.7)	714 (16.8)	2836 (9.4)	4496 (9.8)
Deprivation (IMD)					
1 (Least deprived)	15 368 (17.3)	1 (3.3)	550 (13.2)	5587 (16.2)	9230 (18.4)
2	16 250 (18.3)	1 (3.3)	625 (15.0)	6014 (17.5)	9610 (19.1)
3	17 730 (19.9)	3 (10.0)	797 (19.1)	6675 (19.4)	10 255 (20.4)
4	18 031 (20.3)	8 (26.7)	952 (22.8)	7180 (20.8)	9891 (19.7)
5 (Most deprived)	21 556 (24.2)	17 (56.7)	1258 (30.1)	9007 (26.1)	11 274 (22.4)
Ever smoked	33 240 (66.9)	13 (82.6)	1630 (67.1)	12 920 (67.5)	18 677 (66.4)
Diabetes	11 580 (14.0)	4 (13.8)	593 (14.4)	4188 (13.2)	6795 (14.5)
Hypertension	33 645 (42.9)	10 (90.9)	1672 (46.7)	12 883 (43.6)	19 080 (42.2)
Dyslipidaemia	25 195 (33.0)	11 (91.7)	1249 (36.9)	9826 (34.3)	14 109 (31.9)
Family history of CAD	25 885 (36.9)	0	11 221 (39.4)	10 328 (39.1)	14 336 (35.2)
Previous angina	9683 (12.6)	0	510 (15.1)	4047 (13.9)	5126 (11.5)
Previous MI	9507 (6.7)	2 (6.7)	500 (13.2)	3799 (12.8)	5255 (11.7)
Previous PCI	5933 (7.6)	1 (3.3)	299 (7.9)	2320 (7.9)	3313 (7.4)
Previous CABG	1877 (2.4)	1 (3.3)	97 (2.5)	754 (2.6)	1025 (2.3)
Chronic heart failure	995 (1.3)	0	60 (1.8)	336 (1.2)	599 (1.3)
Cerebrovascular disease	3492 (4.6)	2 (66.7)	147 (4.4)	1353 (4.7)	1990 (4.5)
Renal failure	1607 (2.1)	0	81 (2.4)	644 (2.2)	882 (2.0)
Peripheral vascular disease	2195 (2.9)	3 (75.0)	123 (3.8)	830 (2.9)	1239 (2.8)
Chronic lung disease	8068 (10.5)	0	342 (10.5)	3030 (10.5)	4696 (10.5)
Distance to hospital in km					
0–5	17 421 (19.6)	10 (33.3)	1449 (34.8)	7535 (21.9)	8427 (16.8)
6–15	33 321 (37.4)	19 (63.4)	1829 (43.9)	13 942 (40.4)	17 531 (34.9)
16–29	24 060 (27.0)	1 (3.3)	596 (14.3)	8330 (24.2)	15 133 (30.1)
+30	14 189 (15.9)	0	294 (7.1)	4686 (13.6)	9209 (18.3)
*Trust characteristics (n=84)*					
Total number of beds					
<700	214 (31.4)	–	69 (31.1)	74 (32.2)	71 (30.9)
700–999	246 (36.1)	–	79 (35.6)	78 (33.9)	89 (38.7)
≥1000	222 (32.5)	–	74 (33.3)	78 (33.9)	70 (30.4)
Number of acute and general beds					
<700	292 (43.7)	–	112 (51.4)	89 (39.9)	91 (39.9)
700–999	216 (32.3)	–	60 (27.5)	71 (31.8)	85 (37.3)
≥1000	161 (24.1)	–	46 (21.0)	63 (28.3)	52 (22.8)
Number of in-house interventional cardiologists					
None	208 (28.8)	–	178 (75.7)	24 (9.8)	6 (2.5)
1–5	320 (44.4)	–	39 (16.6)	144 (59.0)	137 (56.6)
>5	193 (26.8)	–	18 (7.7)	76 (31.2)	99 (40.9)
Number of visiting interventional cardiologists					
None	432 (59.9)	–	203 (86.4)	124 (50.8)	105 (43.4)
1–5	199 (27.6)	–	21 (8.9)	80 (32.8)	98 (40.5)
>5	90 (12.5)	–	11 (4.7)	40 (16.4)	39 (16.1)
24/7 PPCI service	261 (36.2)	–	17 (7.2)	106 (43.4)	138 (57.0)
Teaching status	327 (45.4)	–	107 (45.5)	110 (45.1)	110 (45.5)

CABG, coronary artery bypass graft surgery; CAD, coronary artery disease; IMD, index of multiple deprivation; MI, myocardial infarction; PCI, percutaneous coronary intervention; PPCI, primary PCI.

### Variation

**Figure 2:**
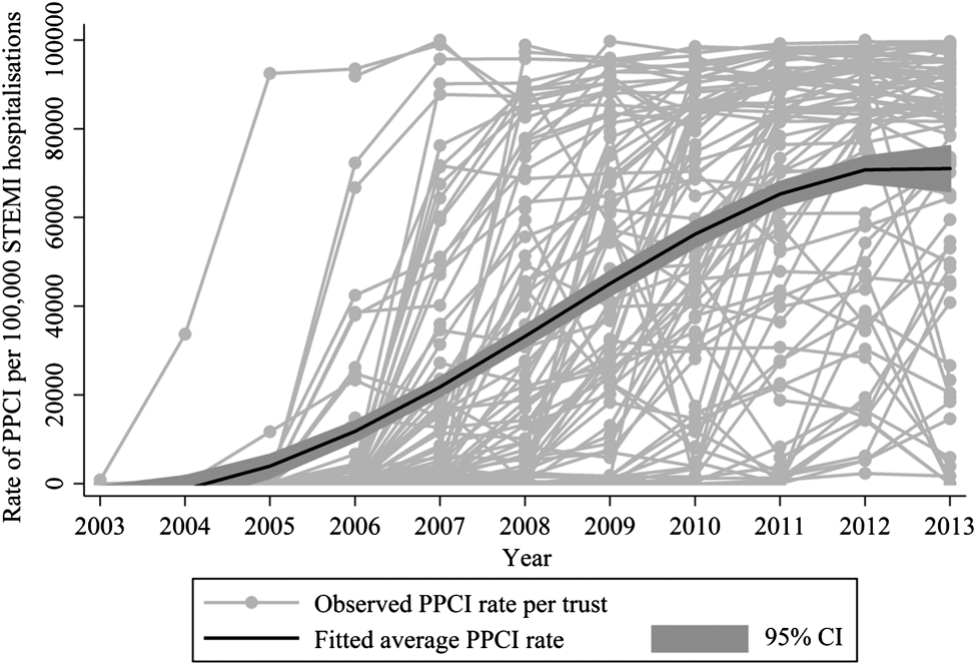
Observed and fitted average rate of primary percutaneous coronary intervention (PPCI) in England from 2003 to 2013.

There was an increase in the utilisation of PPCI over the 10 year study period, with the overall age and sex standardised rate of PPCIs out of all STEMI hospitalisations increasing significantly from 0.01% (95% CI 0.001% to 0.06%) in 2003 to 86.3% (95% CI 79.5% to 94.2%) in 2013 (figure 2). On average, there was a year-on-year increase in PPCI utilisation of 30% (IRR 1.30, 95% CI 1.23% to 1.36%) after adjustment for patient and hospital characteristics. The rate of change varied significantly between hospitals (random effect parameter for year SD=0.22, 95% CI 0.19 to 0.26). The rate of PPCI utilisation ranged from a 4% increase to a threefold increase (IRR 1.04, 95% CI 1.23 to 1.36; and IRR 3.17, 95% CI 1.23 to 1.36) for the central 95% of trusts. The rate of change varied over the study period, with an annual increase in PPCI utilisation of 63% occurring in 2006/2008 (IRR 1.63, 95% CI 1.57 to 1.71) followed by a 9% increase in 2009/2011 (IRR 1.09, 95% CI 1.08 to 1.09) and no further increase in 2012/2013 (IRR 1.01, 95% CI 0.98 to 1.03).

### Determinants of utilisation

There were significant associations between PPCI utilisation and year of procedure, age, previous myocardial infarction, angina, PCI, chronic renal failure, heart failure, diabetes, cerebrovascular disease, distance to hospital, number of hospital beds, number of interventional cardiologists, number of visiting cardiologists, and the availability of a 24/7 PPCI service (table 2). PPCI utilisation was significantly lower for patients with previous myocardial infarction (IRR 0.95, 95% CI 0.93 to 0.98), heart failure (IRR 0.86, 95% CI 0.81 to 0.92), angina (IRR 0.96, 95% CI 0.94 to 0.98), diabetes (IRR 0.97, 95% CI 0.95 to 0.99), chronic renal failure (IRR 0.89, 95% CI 0.85 to 0.94), cerebrovascular disease (IRR 0.96, 95% CI 0.93 to 0.99), aged >80 years (IRR 0.87, 95% CI 0.85 to 0.90), and travel distances >30 km (IRR 0.95, 95% CI 0.93 to 0.98). PPCI utilisation was significantly higher for patients with previous PCI (IRR 1.09, 95% CI 1.05 to 1.12), among trusts with >5 interventional cardiologists (IRR 1.30, 95% CI 1.25 to 1.34), more visiting cardiologists (1–5 consultants: IRR 1.31, 95% 1.26 to 1.36; >6 consultants: IRR 1.42, 1.35 to 1.49), and a 24/7 service (IRR 2.69, 95% CI 2.58 to 2.81). Larger hospitals had lower rates of PPCI (>1000 vs <700 beds, IRR 0.92, 95% CI 0.88 to 0.97). We found that 49% of the unexplained variation in PPCI utilisation was due to differences between trusts (ICC 0.49).

**Table 2:** Unadjusted and adjusted IRRs with 95% CIs assessing variation in utilisation of PPCI in England, 2003–2013

	Unadjusted		Adjusted	
	IRR (95% CI)	p Value	IRR (95% CI)	p Value
Year	1.28 (1.27 to 1.28)	<0.001	1.30 (1.23 to 1.36)	<0.001
Age				
<55	Ref		Ref	
55–64	0.97 (0.96 to 0.99)	<0.001	1.00 (0.98 to 1.01)	0.731
65–79	0.93 (0.92 to 0.95)	<0.001	0.99 (0.97 to 1.01)	0.117
80+	0.83 (0.81 to 0.85)	<0.001	0.87 (0.85 to 0.90)	<0.001
Male gender	1.06 (1.05 to 1.08)	<0.001	1.02 (1.00 to 1.03)	0.044
Distance to hospital in km				
<6	Ref		Ref	
6–15	1.15 (1.13 to 1.18)	<0.001	1.02 (0.99 to 1.04)	0.078
16–29	1.32 (1.28 to 1.35)	<0.001	1.03 (1.00 to 1.05)	0.025
30+	1.28 (1.25 to 1.32)	<0.001	0.95 (0.93 to 0.98)	0.001
Family history of chronic heart disease	1.21 (1.19 to 1.23)	<0.001	1.01 (1.00 to 1.03)	0.085
Previous acute MI	0.92 (0.90 to 0.94)	<0.001	0.95 (0.93 to 0.98)	<0.001
Previous chronic cardiac failure	0.76 (0.72 to 0.81)	<0.001	0.86 (0.81 to 0.92)	<0.001
Previous angina	0.86 (0.84 to 0.88)	<0.001	0.96 (0.94 to 0.98)	0.001
Diabetes mellitus	1.00 (0.98 to 1.02)	0.860	0.97 (0.95 to 0.99)	0.001
Chronic renal failure	0.90 (0.86 to 0.95)	<0.001	0.89 (0.85 to 0.94)	<0.001
Previous PCI	1.11 (1.09 to 1.14)	<0.001	1.09 (1.05 to 1.12)	<0.001
Previous CABG	0.93 (0.89 to 0.97)	<0.001	0.96 (0.92 to 1.01)	0.130
Cerebrovascular disease	0.94 (0.91 to 0.97)	<0.001	0.96 (0.93 to 0.99)	0.012
Smoker	1.04 (1.03 to 1.06)	<0.001	1.01 (0.99 to 1.02)	0.205
Total number of beds at trust				
<700	Ref		Ref	
700–999	0.93 (0.89 to 0.98)	0.003	0.99 (0.95 to 1.04)	0.802
1000+	0.75 (0.71 to 0.79)	<0.001	0.92 (0.88 to 0.97)	0.001
Number of interventional cardiologists				
<5	Ref		Ref	
>5	4.10 (4.00 to 4.21)	<0.001	1.30 (1.25 to 1.34)	<0.001
Number of visiting interventional cardiologists				
0	Ref		Ref	
1–5	3.46 (3.37 to 3.56)	<0.001	1.31 (1.26 to 1.36)	<0.001
6+	4.22 (4.09 to 437)	<0.001	1.42 (1.35 to 1.49)	<0.001
PPCI service				
Daytime only (09:00–17:00)	Ref		Ref	
24 h service 7 days a week	7.19 (6.99 to 7.39)	<0.001	2.69 (2.58 to 2.81)	<0.001
Teaching status (university hospital)	1.27 (1.19 to 1.36)	<0.001	1.01 (0.93 to 1.10)	0.809

CABG, coronary artery bypass graft surgery; IMD, index of multiple deprivation; IRR, incidence rate ratio; MI, myocardial infarction; PCI, percutaneous coronary intervention; PPCI, primary PCI.

## Discussion

This is the first patient-level single country analysis of the temporal implementation of PPCI for the emergency management of STEMI. Over a 10 year study period, we found evidence for an 8 year implementation phase that achieved overall high rates of PPCI per STEMI coverage across England. However, this occurred after a lag of several years. At the end of the study period we found persisting wide variation in rates of PPCI at hospitals and evidence that older and sicker patients were less likely to receive PPCI. While PPCI utilisation was significantly associated with patient-level and trust-level characteristics, one half of the unexplained variation in PPCI utilisation was due to between-hospital factors.

To date, international registry data have shown that implementation of reperfusion therapy for STEMI is insufficient. Earlier studies described how England differed from other European countries, with much slower PPCI adoption,^23^ and greater between provider variation in 30 day mortality.^24^ This study adds more detail—revealing significant provider variation in emergency care. Our findings are surprising since the acute treatment of STEMI in a healthcare setting of universal coverage should result in low inequalities in access. Moreover, for STEMI there is little controversy or ambiguity concerning the effectiveness of PPCI (STEMI is more readily diagnosable than NSTEMI and the majority of patients are hospitalised), and the demand for hospitalisation and treatment of STEMI is closely determined by its incidence.

Provider variation was large during the early PPCI implementation phase—with greater uniformity later. Such diffusion patterns are seen where there is a rapid improvement in treatments or where the range of treatment options has been enhanced,^8^ ^9^ ^13^ ^25^ as in the UK with the transition from fibrinolysis to PPCI. Yet, we identified between-provider variation at the end of the study, when across the health system the majority of STEMI patients received PPCI. Following the implementation phase, between-provider variation in guideline-indicated care should be minimal, and is likely to impact directly on population health outcomes.^13^ ^26^

Variation in healthcare utilisation rates may reflect differences in population need for healthcare—with more services needed in areas of higher demand. Our study exploited rich and representative population panel data including individual-level health measures as well as socioeconomic background information, and temporal and spatial population data. To the contrary, we found PPCI rates were lower at hospitals with a higher proportion of patients who were elderly, diabetic, and with chronic renal and heart failure. This ‘risk-treatment paradox’ has been described for other cardiovascular diseases and contrasts with the absolute risk of a life-threatening complication from an evidence based therapy being unlikely to exceed the survival benefit when applied in high-risk populations.^27^ ^28^ Utilisation of PPCI did not appear to be influenced by area deprivation.

While age and comorbid status contributed to PPCI utilisation, almost half of the unexplained variation in PPCI use was at the hospital level. Supply factors, such as numbers of cardiologists and availability of a 24 h, 7-days-a-week PPCI service were strong determinants of PPCI utilisation. Others have also shown that provider variation is mainly associated with the medical care systems and less with regional differences in disease incidence.^25^ Since PPCI is highly dependent on the availability of specialist resources, our findings are not surprising.^29^ ^30^ Notably the regulation of specialist capacity to perform medical procedures, with the availability of catheterisation laboratories (supplier-induced demand), is a strong determinant of technology use.^8^ ^9^ ^29^ Our study reveals that universal healthcare coverage and evidence for the benefits of a new technology associated with significant improvements in care for a wide sector of the population does not guarantee similar utilisation rates for treatment across a nation. Indeed, our observation of between-trust variation in PPCI use suggests an opportunity to further increase the utilisation of PPCI services and, ultimately, reduce premature cardiovascular deaths. This is particularly pertinent as, a priori, national implementation of PPCI across the NHS was based on a Department of Health directive and funding aiming for equitable PPCI delivery across hospital trusts. To address this shortfall, our findings provide evidence to suggest that PPCI-capable hospitals must have an ‘all-comers’ policy that is compliant with clinical guidelines, provide a 24 h, 7-days-a-week service, be adequately staffed with specialists, and not be so large that there are diseconomies of scale. Future research is needed to ascertain whether the availability of specialist facilities and regulation of capacity explains the large and significant between-hospital variation which was not attributable to case mix and staffing levels.

### Limitations

This study analysed patient-level data aiming specifically to not introduce an ecological fallacy; it does, however, have limitations. While MINAP data entry is subject to routine error checking and a mandatory annual data validation exercise, the analyses were dependent upon the accuracy of the data. We were unable to include patients in non-PPCI capable hospitals, within which we identified a small proportion of patients who did receive PPCI (n=8862; 9%). We controlled for a number of patient risk factors deemed clinically relevant, which are assumed to be exogenous to the hospital, and can be derived from MINAP. However, we do not claim that this set of control variables is exhaustive and the issues of additional confounding are apparent. Even so, a strength of our study is that we used detailed registry data to control for patients’ comorbid conditions, thus making our inferences more robust than using routinely available administrative or survey sample data. Finally, observational analyses such as these reveal important associations, but cannot prove causation.

### Conclusions

This nationwide 10 year study of the uptake and utilisation of PPCI for STEMI found evidence for an 8 year implementation phase that achieved overall high rates of PPCI per STEMI hospitalisation. However, at the end of the implementation phase, there remained wide variation in hospital rates of PPCI. In particular, older and sicker patients were less likely to receive PPCI. While the use of PPCI was significantly associated with patient-level characteristics, there remained significant variation due to hospital-specific factors that were not attributable to differences in medical staffing levels. Compliance with agreed clinical pathways for patients presenting with STEMI is needed to ensure more equitable quality of care.

## Disclosures

All authors have completed the ICMJE uniform disclosure form at http://www.icmje.org/coi_disclosure.pdf and declare no support from any organisation for the submitted work; no financial relationships with any organisations that might have an interest in the submitted work in the previous 3 years; and no other relationships or activities that could appear to have influenced the submitted work.

**Key messages**

**What is already known on this subject?**

- ST-elevation myocardial infarction (STEMI) is a common and major cause of premature death. Timely primary percutaneous intervention (PPCI) is the preferred mode of revascularisation because it is associated with significantly improved outcomes.
- There is variation in the level of use of PPCI which is associated with supply and demand factors, but this has not been investigated at the level of the provider using patient-level data within a single healthcare system.

**What might this study add?**

- While the rates of PPCI increased rapidly (0.01% to 86.3%), the entire implementation took 8 years. Sicker patients were less likely to receive PPCI by 5%, 4% and 3% for those with previous myocardial infarction, angina and diabetes, respectively. We found evidence for wide variation between hospitals in their utilisation of PPCI, and although medical staffing levels were associated with this variation, it did not account for all of the between-hospital variation.

**How might this impact on clinical practice?**

- Our observation of between-trust variation in PPCI use suggests an opportunity to further increase the utilisation of PPCI services and, ultimately, reduce premature cardiovascular deaths. Compliance with agreed clinical pathways for patients presenting with STEMI is needed to ensure more equitable quality of care.

## Supplementary Material

Web supplement
