# Supplementary material for: Patient and hospital determinants of primary percutaneous coronary intervention in England, 2003–2013
Source: Heart. 2016 Jan 6;102(4):313–9. doi: 10.1136/heartjnl-2015-308616 (PMC4752647; doi:10.1136/heartjnl-2015-308616)
Supplement: Web supplement [file heartjnl-2015-308616-s1.pdf]

Appendix

Patient and hospital determinants of primary percutaneous coronary intervention in England, 2003-13

Section 1: Supporting References for Methods

Euclidean Distance

Although Euclidian distance may be underestimated when compared with road based measures, without knowledge of people's mode of transport and whether or not their journey was from home, Euclidean distance provides a good approximation of the proximity of the patient to the hospital.[1]

Intraclass Correlation Coefficient (ICC)

To assess the degree of variation in PPCI utilisation rates attributed to the trust level (and not explained by the model), we obtained an approximation to the intraclass correlation coefficient (ICC) from a multilevel linear regression model which used PPCI rates as the outcome variable, rather than from a multilevel Poisson model for which the calculation of the ICC is not trivial, particularly after multiple imputation.[2] Model estimates from both models were compared for consistency.

Section 2: Multiple Imputation

Table S1. Imputation model specification.

Multiple imputation by chained equations (MICE) was used to generate 10 imputed datasets. The imputation strategy details are provided in the table below.

Variable	Variable Type	Imputation method
Age	Continuous, non-normal	Predictive mean matching
Index of multiple deprivation score	Continuous, non-normal	Predictive mean matching
Ethnicity	Categorical	Polytomous regression
Sex	Binary	Logistic regression
Distance to hospital	Continuous, non-normal	Predictive mean matching
Total beds at hospital	Continuous, non-normal	Predictive mean matching
Number of interventional cardiologists	Continuous, non-normal	Predictive mean matching
PPCI Service 24 hours a day versus daytime only	Binary	Logistic regression
Number of visiting cardiologists	Continuous, non-normal	Predictive mean matching
Diabetes Mellitus	Binary	Default imputation (missing set to no).
Previous hypertension	Binary	Default imputation (missing set to no).
Previous hypercholesterolaemia	Binary	Default imputation (missing set to no).
Previous MI	Binary	Default imputation (missing set to no).
Previous angina	Binary	Default imputation (missing set to no).
Peripheral vascular disease	Binary	Default imputation (missing set to no).
Cerebrovascular disease	Binary	Default imputation (missing set to no).
Chronic obstructive pulmonary disease/Asthma	Binary	Default imputation (missing set to no).
Congestive renal failure	Binary	Default imputation (missing set to no).
Congestive cardiac failure	Binary	Default imputation (missing set to no).
Previous PCI	Binary	Default imputation (missing set to no).
Previous CABG	Binary	Default imputation (missing set to no).
Family history of chronic heart disease	Binary	Default imputation (missing set to no).
Smoking status	Binary	Default imputation (missing set to no).
Primary PCI	Continuous	No imputation required - outcome/auxiliary variable
Year	Continuous	No imputation required - predictor variable

Table S2. Complete case analysis and multiple imputation analysis of adjusted incidence rate ratios (IRRs) with 95% confidence intervals assessing the variation in utilisation of primary percutaneous coronary intervention (PPCI) by patient and trust level factors. England, 2003-2013.

	Complete Cases		Multiple Imputed Data	
	**IRR (95% CI)**	**P-value**	**IRR (95% CI)**	**P-value**
**Year**	1.13 (1.12-1.13)	<0.001	1.12 (1.11-1.12)	<0.001
**Age**				
<55	Ref		Ref	
55-64	1.00 (0.98-1.02)	0.766	1.00 (0.98-1.01)	0.705
65-79	0.99 (0.97-1.01)	0.498	0.99 (0.97-1.01)	0.276
80+	0.89 (0.87-0.92)	<0.001	0.88 (0.86-0.90)	<0.001
**Male gender**	1.02 (1.00-1.03)	0.038	1.02 (1.00-1.03)	0.044
**Distance to hospital in km**				
<6	Ref		Ref	
6-15	1.02 (1.00-1.04)	0.123	1.01 (0.99-1.03)	0.192
16-29	1.02 (0.99-1.04)	0.187	1.02 (1.00-1.05)	0.037
30+	0.95 (0.92-0.97)	<0.001	0.96 (0.93-0.98)	0.001
**Family history of chronic heart disease**	1.02 (1.00-1.03)	0.054	1.01 (1.00-1.03)	0.085
**Previous acute myocardial infarction**	0.96 (0.93-0.98)	0.001	0.95 (0.93-0.98)	<0.001
**Previous chronic cardiac failure**	0.85 (0.79-0.91)	<0.001	0.86 (0.80-0.91)	<0.001
**Previous angina**	0.97 (0.94-0.99)	0.009	0.96 (0.94-0.98)	0.001
**Diabetes mellitus**	0.96 (0.94-0.99)	0.001	0.96 (0.94-0.98)	<0.001
**Chronic renal failure**	0.91 (0.86-0.96)	<0.001	0.90 (0.86-0.95)	<0.001
**Previous PCI**	1.09 (1.05-1.12)	<0.001	1.09 (1.05-1.12)	<0.001
**Previous CABG**	0.96 (0.92-1.01)	0.124	0.96 (0.92-1.01)	0.130
**Cerebrovascular disease**	0.97 (0.93-1.00)	0.082	0.96 (0.93-0.99)	0.012
**Smoker**	1.01 (1.00-1.03)	0.094	1.01 (0.99-1.02)	0.205
**Total number of beds at hospital**				
<700	Ref		Ref	
700-999	0.90 (0.85-0.94)	<0.001	0.99 (0.95-1.04)	0.802
1000+	0.86 (0.81-0.92)	<0.001	0.92 (0.88-0.97)	0.001
**Number of interventional cardiologists**				
<5	Ref		Ref	
>5	1.28 (1.24-1.33)	<0.001	1.35 (1.31-1.39)	<0.001
**Number of visiting cardiologists**				
0	Ref		Ref	
1-5	1.41 (1.36-1.46)	<0.001	1.39 (1.35-1.44)	<0.001
6+	1.58 (1.51-1.65)	<0.001	1.42 (1.36-1.48)	<0.001
**PPCI offer**				
Daytime only (9-17)	Ref		Ref	
24 hours service 7 days a week	2.85 (2.74-2.96)	<0.001	2.59 (2.50-2.68)	<0.001
**Hospital teaching status (university hospital)**	1.09 (0.99-1.21)	0.074	1.01 (0.93-1.10)	0.818

Abbreviations: CABG – Coronary artery bypass graft; IMD – Index of multiple deprivation; IRR – Incidence rate ratio; PPCI – Primary percutaneous coronary intervention

Section 3: Overdispersion

Overdispersion was checked by comparing a Poisson and negative binomial regression model using the Akaike's information Criterion (AIC) and Bayesian Information Criterion (BIC). Poisson regression was chosen in favour of negative binomial regression as the AIC and BIC were minimised for the Poisson model.

Table S3: Model comparison of final Poisson and negative binomial models after imputation

Model	AIC	BIC
Poisson model	225576.7	225834
Negative binomial model	225635	225892.3

References

1 Shahid R, Bertazzon S, Knudtson ML, et al. Comparison of distance measures in spatial analytical modeling for health service planning. *BMC health services research* 2009;**9**:200.

2 Goldstein H, Browne W, Rasbash J. Partitioning variation in multilevel models. *Understanding Statistics: Statistical Issues in Psychology, Education, and the Social Sciences* 2002;**1**:223-31.
